# Polygenic risk-based prediction of heart failure in young patients with atrial fibrillation

**DOI:** 10.1093/europace/euaf138

**Published:** 2025-09-05

**Authors:** Parichatra Homhuan, Manpreet Kour

**Affiliations:** Department of Cardiology, Royal Free Hospital, Pond St, London NW3 2QG, UK; Department of Cardiology, Royal Free Hospital, Pond St, London NW3 2QG, UK

The recent study by Ahn *et al.*^1^ provides compelling evidence that polygenic risk scores (PRS) can substantially improve the prediction of incident heart failure (HF) in patients with atrial fibrillation (AF), particularly among younger individuals who may benefit most from early intervention. Their work meaningfully contributes to the ongoing efforts to personalize cardiovascular care through genomic risk stratification.

However, the study does not yet propose the crucial actionable thresholds or clinical pathways necessary for the practical implementation of PRS. While the authors demonstrate added predictive value, there remains a critical lack of guidance on when a PRS should be considered ‘high enough’ to warrant specific interventions, nor do they offer a framework for integrating this genetic risk information with established clinical tools such as CHA_2_DS_2_-VASc or guideline-based care models.

This operational gap significantly hinders clinicians from being able to move actionable genetic risk insights into concrete, structured care plans. Lacking well-defined decision points, even statistically valid tools risk stagnation at the edge of implementation.

Therefore, future research must prioritize the definition of quantitative PRS cut-offs linked to specific clinical actions (e.g. guiding decisions on anticoagulation intensity, frequency of cardiac monitoring, or patient selection for early rhythm control strategies) and rigorously evaluate these frameworks through prospective validation or randomized controlled trials.

Such work will be essential to demonstrate real-world impact on patient outcomes and clinical decision-making.

Ahn *et al.* have laid a strong scientific foundation. The next challenge is to build the clinical framework that enables this genomic insight to deliver actionable benefit in routine AF care.
